# The Exopolysaccharide Cepacian Plays a Role in the Establishment of the *Paraburkholderia phymatum* – *Phaseolus vulgaris* Symbiosis

**DOI:** 10.3389/fmicb.2020.01600

**Published:** 2020-07-16

**Authors:** Yilei Liu, Barbara Bellich, Sebastian Hug, Leo Eberl, Paola Cescutti, Gabriella Pessi

**Affiliations:** ^1^Department of Plant and Microbial Biology, University of Zurich, Zurich, Switzerland; ^2^Department of Life Sciences, University of Trieste, Trieste, Italy

**Keywords:** *Rhizobium*, legume, nodulation, *Burkholderia cepacia* exopolysaccharide (*bce*) cluster, biofilm, nitrogen limitation

## Abstract

*Paraburkholderia phymatum* is a rhizobial strain that belongs to the beta-proteobacteria, a group known to form efficient nitrogen-fixing symbioses within root nodules of several legumes, including the agriculturally important common bean. The establishment of the symbiosis requires the exchange of rhizobial and plant signals such as lipochitooligosaccharides (Nod factors), polysaccharides, and flavonoids. Inspection of the genome of the competitive rhizobium *P. phymatum* revealed the presence of several polysaccharide biosynthetic gene clusters. In this study, we demonstrate that *bceN*, a gene encoding a GDP-D-mannose 4,6-dehydratase, which is involved in the production of the exopolysaccharide cepacian, an important component of biofilms produced by closely related opportunistic pathogens of the *Burkholderia cepacia* complex (*Bcc*), is required for efficient plant colonization. Wild-type *P. phymatum* was shown to produce cepacian while a *bceN* mutant did not. Additionally, the *bceN* mutant produced a significantly lower amount of biofilm and formed less root nodules compared to the wild-type strain with *Phaseolus vulgaris* as host plant. Finally, expression of the operon containing *bceN* was induced by the presence of germinated *P. vulgaris* seeds under nitrogen limiting conditions suggesting a role of this polysaccharide in the establishment of this ecologically important symbiosis.

## Introduction

Rhizobia are phylogenetically diverse soil bacteria, which possess the ability to infect the roots of certain legumes and induce nodules on roots or stems. The nodules represent specialized root organs in which rhizobia fix atmospheric nitrogen (N_2_) into ammonia, thereby giving legumes a pronounced growth advantage in nitrogen (N) deprived soils (Masson-Boivin et al., 2009; Oldroyd et al., 2011; Udvardi and Poole, 2013). Until recently, rhizobia were thought to belong exclusively to the alpha-subclass of proteobacteria (alpha-rhizobia, isolated first in 1888 by Beijerinck). This situation changed in 2001, when two genera belonging to the beta-proteobacteria (*Burkholderia* and *Cupriavidus*) were discovered to form nitrogen-fixing root nodules (beta-rhizobia) (Chen et al., 2001; Moulin et al., 2001; Bontemps et al., 2010). For the legume nodulating strains that belong to the environmental clade of the genus *Burkholderia*, the new genus *Paraburkholderia* has been recently proposed (Sawana et al., 2014; Beukes et al., 2017). *Paraburkholderia* strains have originally been isolated from South American mimosoid and South African papillionoid legumes (Chen et al., 2003, 2005; Elliott et al., 2007; dos Reis et al., 2010; Mishra et al., 2012; Bournaud et al., 2013; Lemaire et al., 2015, 2016). The diversity of mimosoid-nodulating *Paraburkholderia* is high and includes species such as *P. phymatum*, which is promiscuous and highly competitive against other alpha- and beta-rhizobial strains for nodulating several mimosoid and papilionoid legumes of major agricultural importance including the common bean (*Phaseolus vulgaris*) (Elliott et al., 2009; Talbi et al., 2010; Melkonian et al., 2014; Moulin et al., 2014; Lardi et al., 2017a). The establishment of a rhizobia-legume nitrogen-fixing symbiosis is a highly complex and regulated process involving the exchange of a series of specific bacterial and plant signals. Among these signals, bacterial lipochitooligosaccharides (Nod factors) and surface polysaccharides as well as flavonoids secreted by plant roots play crucial roles (Masson-Boivin et al., 2009; Downie, 2010). Rhizobia produce a large variety of surface polysaccharides such as exopolysaccharides, outer membrane-localized lipopolysaccharide (LPS), capsule polysaccharide (CPS), K-antigen polysaccharide (KPS), cyclic beta-glucans, and glucomannan. LPS as well as exopolysaccharides, KPS, and cyclic glucans are important for the interaction with the host plant (Breedveld and Miller, 1994; Cheng and Walker, 1998; Niehaus and Becker, 1998; Pellock et al., 2000; Fraysse et al., 2003; Laus et al., 2006; Skorupska et al., 2006; Jones et al., 2007; Marczak et al., 2017). Exopolysaccharides are secreted into the root environment and are generally only weakly associated with the bacterial surface. They are species- or strain-specific and have heterogeneous structures containing different monosaccharides (D-glucose, D-galactose, D-mannose, L-rhamnose, D-glucuronic acid, and D-galacturonic acid) that are linked in a linear or branched structure. The degree of polymerization, the type of glycosidic bonds as well as the variety of the non-carbohydrate modifications (e.g., acetyl, pyruvyl, or succinyl groups) also greatly contribute to the diversity of bacterial exopolysaccharide matrices. Alpha-rhizobial exopolysaccharide molecules have been shown to be important for the development of an effective symbiosis (Leigh et al., 1985; Djordjevic et al., 1987; Battisti et al., 1992; Urzainqui and Walker, 1992; Gonzalez et al., 1996; Pellock et al., 2000). Exopolysaccharide-deficient mutants of rhizobia that infect plants producing indeterminate-type nodules (e.g., *Rhizobium leguminosarum* and *Sinorhizobium meliloti*) were compromised in their ability to induce root hair curling and to form infection-threads (Leigh et al., 1985; Cheng and Walker, 1998; Niehaus and Becker, 1998; Pellock et al., 2000). In addition, exopolysaccharides represent the major component of the biofilm matrix and are thus important for bacterial attachment to surfaces, protection against environmental stresses and antimicrobial compounds, and evasion of host defense responses (Santos et al., 2001; Downie, 2010; Ferreira et al., 2010, 2011; Geddes et al., 2014; Maroti et al., 2015; Arnold et al., 2018). Some polysaccharides have also been shown to suppress plant defense responses and to be involved in plant signaling by binding to specific membrane-spanning receptor-like proteins with significant similarity to Nod factor receptor 1 (NFR1) (Kawaharada et al., 2015). In beta-rhizobia such as the *Paraburkholderia* strains, nothing is known about the requirement of specific polysaccharides for the establishment of a successful symbiosis with legumes. However, it was shown that several *Burkholderia* and *Paraburkholderia* species, including *P. phymatum*, produce the polysaccharide cepacian (CEP) (Ferreira et al., 2010). In *Burkholderia* strains of the *Burkholderia cepacia* complex (*Bcc*), this is the main exopolysaccharide produced (Cescutti et al., 2000; Conway et al., 2004; Ferreira et al., 2007; Cuzzi et al., 2014). In this group of opportunistic pathogens that can cause lung infections in cystic fibrosis (CF), immunocompromised or granulomatous disease patients, CEP was associated with bacterial persistence in the lung (Conway et al., 2004; Cunha et al., 2004; Sousa et al., 2007; Zlosnik et al., 2011; Ferreira et al., 2019) and shown to act as a protective barrier against antibiotics and other stresses such as oxidative stress, desiccation, and metal ion stress (Bylund et al., 2006; Benincasa et al., 2009; Ferreira et al., 2010, 2011; Cuzzi et al., 2012). Importantly, many of these potentially pathogenic *Bcc* strains, which produce CEP, are also naturally associated with plants (Coenye and Vandamme, 2003; Eberl and Vandamme, 2016). *B. cenocepacia* for example was originally described as a pathogen of onions and inhabits the soil and rhizosphere of several plants (Burkholder, 1950; LiPuma et al., 2002; Ramette et al., 2005; Dalmastri et al., 2007; Lee and Chan, 2007).

The structure of CEP has been elucidated; it consists of the five monosaccharides D-glucose, D-mannose, D-rhamnose, D-glucuronic acid, and D-galactose in a 1:1:1:1:3 ratio and is modified in the side chains with acetyl groups (Cerantola et al., 1999; Cescutti et al., 2000, 2010). The first step in CEP biosynthesis is the formation of sugar-nucleotide precursors, which are assembled by the action of several glycosyltranferases into a heptasaccharide repeating unit. CEP is acetylated during the repeating unit process at the inner membrane and the number of substitution seems to be strain dependent (Cescutti et al., 2010). The lipid carrier-linked heptasaccharide repeating units are exported across the inner membrane and polymerized in the periplasm by the Wzy-dependent pathway that involves a transport protein called flippase and polysaccharide polymerase, respectively (Moreira et al., 2003; Ferreira et al., 2010).

In *Bcc* members and other animal and plant pathogenic non-*Bcc* isolates, CEP is synthetized by two gene clusters (*bce*-I and *bce*-II, for *B. cepacia* exopolysaccharide), which are separated by 130 to 314 kb (Ferreira et al., 2010). Interestingly, in the genome of environmental strains previously belonging to the *Burkholderia* genus (e.g., *P. phymatum* STM815, *P. xenovorans* LB400, *P. phytofirmans* PsJN, *P. graminis* C4D1M, and *Caballeronia glathei* DSM 50014), the CEP genes are not separated, but are organized as a continuous gene cluster (Moreira et al., 2003; Ferreira et al., 2010, 2019).

In this work, we demonstrate for the first time a role of CEP in the symbiotic interaction of a *Paraburkholderia* strain with its host plant. Through mutagenesis, the *bceN* gene of *P. phymatum*, which encodes a protein with GDP-D-mannose 4,6-dehydratase enzyme activity (Sousa et al., 2013), was shown to be important for CEP production and biofilm formation. Moreover, common bean plants infected with a *P. phymatum bceN* mutant produced fewer nodules on the roots but otherwise performed nitrogen fixation as efficiently as nodules infected with the wild type, suggesting a role of CEP in root attachment, i.e., the first step of establishing a symbiosis. Finally, we show here that expression of the operon containing *bceN* is induced under nitrogen-limiting conditions and in the presence of germinated seeds or root exudates.

## Materials and Methods

### Bacterial Strains, Plasmids, and Growth Conditions

The bacterial strains, plasmids, and primers employed in this work are listed in Supplementary Table S1. *Escherichia coli* and *P. phymatum* strains were routinely maintained on Luria-Bertani (LB) (Miller, 1972) and ABS (AB-minimal medium with 15 mM sodium succinate as carbon source) (15.13 mM (NH_4_)_2_SO_4_, 42.25 mM Na_2_HPO_4_, 22 mM KH_2_PO_4_, 51.33 mM NaCl, 2 mM MgCl_2_, 0.1 mM CaCl_2_, 3 μM FeCl_3_, 15 mM succinate, and 1.5% agar) plate, respectively. *E. coli* cells were cultivated under aerobic conditions in liquid LB medium, whereas *P. phymatum* cells were cultivated in the modified LB-NaCl (LB medium without salt: 10 g tryptone and 5 g yeast extract per liter). Where applicable, the following antibiotic concentrations were used: chloramphenicol (20 μg/ml for *E. coli* and 80 μg/ml for *P. phymatum*), kanamycin (25 μg/ml for *E. coli* and 50 μg/ml for *P. phymatum*). The variations of AB-minimal liquid medium used in this study are ABS (see above), (A)BS that is ABS without nitrogen source (15.13 mM Na_2_SO_4_, 42.25 mM Na_2_HPO_4_, 22 mM KH_2_PO_4_, 51.33 mM NaCl, 2 mM MgCl_2_, 0.1 mM CaCl_2_, 3 μM FeCl_3_, and 15 mM succinate), and (A)B that is ABS without nitrogen and carbon source (15.13 mM Na_2_SO_4_, 42.25 mM Na_2_HPO_4_, 22 mM KH_2_PO_4_, 51.33 mM NaCl, 2 mM MgCl_2_, 0.1 mM CaCl_2_, and 3 μM FeCl_3_). In nitrogen limited conditions, (A)BS medium was supplemented with 0.3 mM NH_4_Cl. To measure biofilm production, the cells were grown in nitrogen limited (A)BM medium, which contains 10 mM mannitol as carbon source.

### Construction of a *P. phymatum bceN* Mutant and Reporter Strains

Chromosomal DNA of *P. phymatum* STM815 was isolated by using GenElute^TM^ Bacterial Genomic DNA Kit (Sigma-Aldrich, St. Louis, MO, United States). Plasmid DNA from *E. coli* strains was obtained by using the QIAprep Spin Miniprep Kit (Qiagen, Hilden, Germany). To generate a *bceN* mutant, an internal fragment of Bphy_1069 was PCR amplified with primers Bphy1069_IM_F_*Eco*RI and Bphy1069_IM_R_*Xba*I, cloned into a pSHAFT2 plasmid between the *Eco*RI and *Xba*I sites. The plasmid pSHAFT-bceN was then transferred into the wild-type *P. phymatum* to generate the Bphy_1069 insertion mutant (*bceN*-IM). Genomic integration of the plasmid was confirmed with PCR using primers Bphy1069_veri_F and pSHAFT_F. The coding sequence of Bphy_1069 was produced by PCR with primers Bphy_1069_comp_F_*Eco*RI and Bphy_1069_comp_R_*Bam*HI and cloned into pBBR1MCS-2 between the *Eco*RI and *Bam*HI sites. The constructed plasmid pBBR1MCS2-*bceN* was transferred into *bceN*-IM to complement the mutant strain, resulting in the complemented strain (*bceN*-COMP). In *bceN*-COMP, *bceN* expression is driven by the *lacZ* promoter. We also transferred the vector pBBR1MCS-2 into *bceN*-IM as an empty vector control strain of *bceN*-COMP (*bceN*-pBBR). In order to quantify the expression of the CEP encoding genes, a promoter fusion strain was constructed. The promoter region of the *bceOVN* operon was cloned using primers bceOVNpro_F_*Xba*I and bceOVNpro_R_*Eco*RI into a pPROBE-NT vector between the *Xba*I and *Eco*RI sites. The resulting plasmid and the empty vector pPROBE-NT were transconjugated into wild-type *P. phymatum* to create the promoter reporter (WT-pPROBE-bceOVN) and the negative control strain (WT-pPROBE), respectively. The correct sequence of the clones was confirmed at Microsynth AG (Balgach, St. Gallen, Switzerland).

### Phenotypical Analysis

*Paraburkholderia phymatum* strains (WT, *bceN*-IM and *bceN*-COMP) were grown aerobically in liquid LB-NaCl and ABS media (three independent cultures; 50 ml liquid in 250 ml Erlenmeyer flasks at 30°C with shaking at 220 rpm). To quantify transcription of the *bceN* operon, the GFP expression of the promoter reporter was recorded in a 96-well plate (Falcon, Corning, United States) by a TECAN plate reader (TECAN Infinite M200 PRO, Tecan Trading AG, Switzerland). Cells of strain WT-pPROBE-bceOVN together with the negative control WT-pPROBE were washed twice with (A)B medium and the OD_600_ was normalized to 0.2 in four different media: ABS, (A)BS supplemented with 0.3 mM NH_4_Cl, in the presence or absence of bean root exudates. In the media containing root exudates, half the volume of the medium was replaced by freshly prepared root exudate solution (final concentration 50% root exudates). Each strain was tested with three independent biological clones in technical duplicates. The cells were added into a 96-well plate, which was then incubated at 30°C for 48 h. OD_600_ and GFP were measured by TECAN plate reader.

Polysaccharide production was assayed as previously described (Lardi et al., 2017b). Three independent clones of each strain were tested on three media containing 0.06% yeast extract, 1.5% Agar, and 1% of one of three different carbon sources (mannitol, glucose, and galactose). The plates were incubated at 30°C for three days before analyzing the results. Biofilm assays were carried out as previously described (Huber et al., 2001) with slight modifications. The cells were grown in nitrogen limited (A)BM medium. The plate was incubated at 30°C for 5 days before OD_550_ (growth) and OD_570_ (crystal violet) measurement.

### Exopolysaccharides Extraction and Nuclear Magnetic Resonance (NMR) Analysis

*Paraburkholderia phymatum* wild-type and mutant strains were cultivated in the modified LB-NaCl liquid medium. Cells were washed in liquid YEM (0.06% yeast extract, 1% mannitol) twice and spread on 9 cm YEM agar plate at the density of OD_600_ 0.1 cells per plate. The plates were incubated at 30°C for 4 days. The bacterial lawn was collected with 0.9% NaCl (about 3 mL per dish), gently stirred at 10°C for 2 h, centrifuged at 22,400 × *g* at 4°C for 30 min to separate the cells from the supernatant, which was subsequently precipitated with four volumes of cold ethanol. The precipitated material was recovered by centrifugation at 1,900 × *g* at 4°C for 30 min and subsequently dried to eliminate the alcohol. The samples were dissolved in 0.1 M NaCl and dialyzed first against 0.1 M NaCl and then against water. Polysaccharides were recovered by lyophilization. ^1^H nuclear magnetic resonance (NMR) spectra were recorded on a 500 MHz VARIAN spectrometer. Polysaccharides (3.7 mg of exopolysaccharide produced by the wild-type strain, 2.6 mg by *bceN*-IM and 2.3 mg *bceN*-COMP) were exchanged two times with 99.9% D_2_O by lyophilization and subsequently dissolved in 0.6 mL of 99.96% D_2_O. ^1^H NMR spectra were recorded at 50°C. Polysaccharides (5.1 mg of exopolysaccharides produced by the wild-type strain, 4.2 mg by *bceN*-IM strain, and 4.7 mg by *bceN*-COMP) were dissolved in 4 mL of water (16 h with stirring) and sonicated using a Branson sonifier equipped with a microtip at 2.8 Å, in order to decrease their molecular masses. The samples were cooled in an ice bath and sonicated using 10 bursts of 1 min each, separated by 1 min intervals. In order to de-acetylate the exopolysaccharide, a solution of NaOH was added to each exopolysaccharide solution to a final NaOH concentration of 0.01 M. The reaction was let to proceed 5 h under a N_2_ flow with stirring, followed by extensive dialysis against water. Afterward, the exopolysaccharide solutions were taken to pH 6.6 and recovered by lyophilization. They were exchanged with 99.9% D_2_O as described above, subsequently dissolved in 0.6 mL of 99.96% D_2_O and subjected to ^1^H NMR spectroscopy at 70°C.

### Plant Infection Test, Root Attachment Assay, and Production of Root Exudates

Common bean seedlings (*Phaseolus vulgaris*, cv. Negro Jamapa) were surface sterilized as previously described (Talbi et al., 2010). Seeds were subsequently placed on 0.8% agar plates and incubated in the dark at 28°C. After 40 h, germinated seedlings were planted into autoclaved yogurt-jars containing vermiculite (VTT-Group, Muttenz, Switzerland) and 170 ml diluted Jensen medium (Hahn and Hennecke, 1984). The bacterial cells of each strain were grown in LB-NaCl liquid medium overnight (15 h) and washed twice in A(B) medium. Then the OD_600_ of each strain was adjusted to 0.025. A total of 1 ml of normalized cells (about 10^7^ cells) were directly inoculated on to each germinated seedling. Important symbiotic properties (nodule number, nodule dry weight, and nitrogenase activity of bacteroids) were determined as described previously (Göttfert et al., 1990). Importantly, nitrogenase activity was normalized with nodule dry weight. The plants were grown with the following parameters: temperature: 22°C at night and 25°C during the day; light: approximately 16 h (200 μM intensity); humidity: 60%. The plants were harvested 21 days post infection (dpi).

In the root attachment assay, germinated beans and bacterial cells to be tested were prepared in the same way as for the plant infection tests. Each bean root with length between 1 and 2.5 cm was submerged in 1 ml bacterial inoculum for 4 h. To remove the loosely attached bacteria, individual beans were briefly rinsed with water and then twice washed in 20 ml PBS-S buffer (130 mM NaCl, 7 mM Na_2_HPO_4_, 3 mM NaH_2_PO_4_, pH 7.0, 0.02% Silwet L-77) with vigorous agitation at 180 rpm for 20 min (Bulgarelli et al., 2012). Afterward, each root was carefully cut off and homogenized with one 7 mm glass bead in 500 μl (A)B medium by a bead mill (TissueLyser II, QIAGEN). The homogenate was then diluted serially and plated on LB-NaCl agar plates. The number of attached cells was determined by colony forming units.

Root exudates were collected in a sterile manner by the following procedure. Bean seeds were germinated as in the plant infection tests. A sterile loose cotton ball was placed on the surface of 30 ml sterile dH_2_O in a 50 ml standing syringe with a stopper. Eight germinated seeds were carefully placed on the cotton ball with roots soaking into the water. Seedlings were incubating at 28°C for 4 days. Root exudate water solution was collected and immediately used in the experiments.

The induction of promoter GFP fusion reporters by germinated bean seeds were visualized on soft agar plates. To do so, bean seeds were surface sterilized and germinated, the reporter strain was cultivated and washed in the same way as in the plant infection tests. The cells were added into the melted soft agar (0.8%) media ABS and (A)BS with 0.3 mM NH_4_Cl to reach a final OD_600_ = 0.1 at approximately 37°C and was immediately poured into a 9-cm petri-dish. Before the plate had solidified, one germinated bean was placed on top of a soft agar medium plate with the root sticking into the medium. The images of the plates were taken every 24 h using a custom built fluorescence imaging device (Infinity 3 camera, Lumenera, Canada). The excitation/emission wavelength of GFP is 490 nm/510 nm.

### Bioinformatics and Statistical Analyses

**FIGURE 1:**
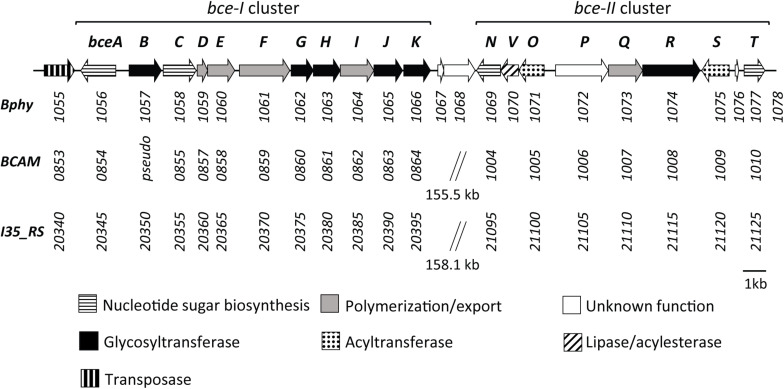
Genetic organization of the *bce* gene clusters in *P. phymatum* STM815 (Bphy) in comparison with *B. cenocepacia J2315* (BCAM), and *B. cenocepacia* H111 (I35_RS). Gene numbers are listed under each gene indicating a sequential arrangement of genes in two *bce* clusters (*bce*-I and *bce*-II), which are either continuous or separated. Functional categorization adapted from Ferreira et al. (2010).

The statistical analyses of biofilm formation, symbiotic properties of bean nodules, root attachment assay, and GFP induction of promoter fusions were performed by GraphPad Prism 7.00 using unpaired *t*-tests (*p*-value: ^****^<0.0001, ^∗∗∗^<0.001, ^∗∗^<0.01, and ^∗^<0.05). For *bce* cluster analysis (Supplementary Figure S4), the DNA sequence from *P. phymatum* STM815 *bce*-I cluster (position: 1183179–1199877) and *bce*-II cluster (1201886–1215931) was searched with blast-2.9.0 + against a self-made blast database. The blast database contained all DNA sequences of the representing genomes on NCBI from the 16S rRNA gene sequences used to create Figure 1 in the published review (Eberl and Vandamme, 2016). For the following five strains *B. arboris* R-24201, *B. seminalis* R24196, *B. tuberum* STM678, *B. unamae* MTI-641, and *B. silvatlantica* SRMrh-20 no reference genome was available on NCBI. The percent of identity was above 72% for each blast result.

## Results

### Genetic Organization of the *bce* Gene Cluster in *P. phymatum*

Previous work has identified the CEP biosynthetic gene cluster in *P. phymatum* (Ferreira et al., 2010). The products of this gene cluster containing 22 genes showed high amino acid identity (from 61 to 85%) with enzymes required for the biosynthesis of the polysaccharide CEP in the opportunistic pathogen *Bcc* strains *B. cenocepacia* H111 and *B. cenocepacia* J2315 (Figure 1). In strains H111, J2315 and other *Bcc* members, the CEP genes are located in two separated clusters (*bce*-I and *bce*-II). In contrast, the genomic organization in *P. phymatum* STM815 differs in that the respective genes were found adjacent to each other forming one continuous cluster (Figure 1) (Ferreira et al., 2010): genes of the *bce*-I cluster (*bceABCDEFGHIJK*, Bphy_1056–Bphy_1066) are followed by the *bce*-II cluster (*bceNVOPQRST*, Bphy_1069–Bphy_1077). While the gene products BceA (Bphy_1056), BceC (Bphy_1058), BceN (Bphy_1069), and BceT (Bphy_1077) are predicted to be involved in sugar-nucleotide biosynthesis, BceB (Bphy_1057), BceG (Bphy_1062), BceH (Bphy_1063), BceJ (Bphy_1065), BceK (Bphy_1066), BceO (Bphy_1071), BceR (Bphy_1074), and BceS (Bphy_1075) are predicted to play a role in heptasaccharide repeat-unit assembly, whereas BceD (Bphy_1059), BceE (Bphy_1060), BceF (Bphy_1061), BceI (Bphy_1064), and BceQ (Bphy_1073) are needed for polymerization and export. Bphy_1070 (*bceV*) codes for a putative lipase, which is missing in the *bce* cluster of pathogenic *Burkholderia* strains and a subgroup of *Paraburkholderia* strains such as *P. tropica*, *P. unamae*, and *P. mimosarum*. The *P. phymatum bce* cluster also contains genes encoding proteins of unknown function, such as Bphy_1072 (*bceP*), Bphy_1076, and Bphy_1067-68. The gene *bceP* is the first in a putative operon (*bcePQR*) and the corresponding protein contains a six-bladed beta-propeller domain that can also be found in TolB proteins. The two genes Bphy_1067-68 are located in a putative operon downstream of *bceK* (Figure 1) and are only present in the phylogenetically closely related *Paraburkholderia* strains *P. caribensis*, *P. hospita*, and *P. terrae*. Bphy_1067 displays homology to transcriptional regulators of the xenobiotic response element (XRE) family that have a helix-turn-helix DNA-binding motif similar to that of the CI repressor and the Cro proteins of λ bacteriophage. Bphy_1068 codes for a hipA (toxin) domain-containing protein and is often co-occurring with Bphy_1067 in the genomes of other *Paraburkholderia* strains.

A comparison with the *bce* genes present in strain J2315 and other *Bcc* strains revealed that two genes, *bceM* and *bceU* were missing in the *P. phymatum* STM815 *bce* cluster. In J2315 and other *Bcc* strains, these two genes are located upstream (*bceM*) and downstream (*bceU*) of the *bce*-II cluster. BceM codes for a GDP-6-deoxy-D-lyxo-4-hexulose reductase (also called RMD) and *bceU* for a membrane protein involved in the acylation of CEP.

Interestingly, upstream of the first gene of the *bce* cluster we identified a gene (Bphy_1055) potentially coding for a transposase A-like protein (Figure 1), which could be involved in the acquisition or transfer of this gene cluster between *Burkholderia* strains.

### Mutation of *bceN* Reduces Exopolysaccharides and Biofilm Production in *P. phymatum*

*BceN* (Bphy_1069) is the last gene of the *bceOVN* operon and codes for a GDP-D-mannose 4,6-dehydratase with 82% identity to the previously characterized BceN of *Bcc* strains H111 and J2315 (Sousa et al., 2013). This enzyme catalyzes the conversion of GDP-D-mannose into the sugar nucleotide GDP-4-keto-6-deoxy-D-mannose, which is the precursor of GDP-D-rhamnose, one of the sugar nucleotides of CEP. Interestingly, two additional *bceN* paralogs have been found in two other potential exopolysaccharides clusters in *P. phymatum* genome (Bphy_6734 and Bphy_2471). Since GDP-D-rhamnose is one important sugar nucleotide required for CEP synthesis, the *bceN* gene of *P. phymatum* was inactivated by the insertion of a suicide plasmid (see section “Materials and Methods”). The *bceN* mutant strain (called *bceN*-IM) was complemented by introducing a pBBR1MCS-2 plasmid carrying the *bceN* gene (*bceN*-COMP). While the *bceN* mutant did not show a growth defect under aerobic conditions in rich (LB without salt) and minimal ABS media, the growth of the complemented strain was slightly slower compared to the wild-type but reached the same final OD_600_ after 11 h of incubation in rich medium (Supplementary Figure S1).

**FIGURE 2:**
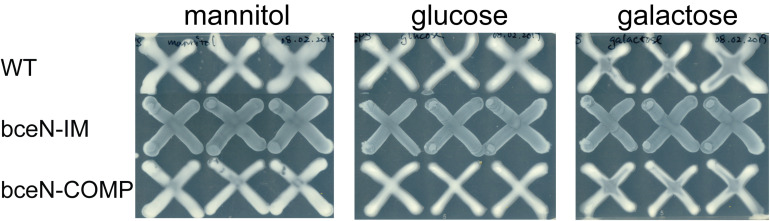
Exopolysaccharide production of *P. phymatum* wild-type (WT), *bceN* mutant (bceN-IM), and complemented (bceN-COMP) strains assayed on plates containing 0.06% yeast extract and 1% of the indicated carbon source. Three independent biological replicates were tested per strain. Plates were incubated at 30°C for 3 days.

In order to evaluate the contribution of *bceN* to exopolysaccharide synthesis, mutant and complemented strain were grown on plates containing different carbon sources (glucose, galactose and the sugar alcohol mannitol) (Figure 2). In contrast to the wild-type and the complemented strain, the appearance of the *bceN* mutant was non-mucoid on all carbon sources tested, suggesting that BceN plays an important role in exopolysaccharide production. The amount of exopolysaccharide produced in the wild-type strain did not seem to vary depending on the carbon sources (Figure 2).

**FIGURE 3:**
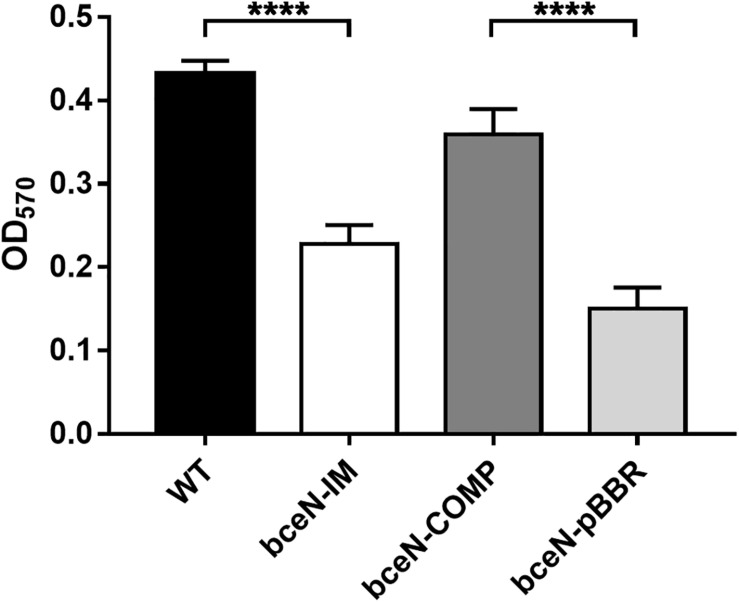
Biofilm production in *P. phymatum* wild-type and mutant strains. The cells were incubated in nitrogen limited (A)BM minimal medium at 30°C for 5 days before biofilm production (OD_570_) was quantified. Three independent biological replicates of each strain were assayed. Wild-type (WT) and the complemented (bceN-COMP) strains produced significantly more biofilm than the mutant (bceN-IM) and the mutant containing the empty vector (bceN-pBBR), respectively. Error bars indicate the standard error of the mean (SEM). ****p-value < 0.0001.

Since exopolysaccharides are key components of the biofilm matrix in numerous bacteria, the capacity of four *P. phymatum* strains (wild-type, *bceN*-IM, *bceN*-COMP, and *bceN*-pBBR) to form biofilm was assessed in 96-well polystyrene plates using the crystal violet method. The cells were grown in minimal medium (A)BM with mannitol as carbon source under nitrogen limiting condition and incubated aerobically for 5 days at 30°C. Under this growth condition, all strains grew to a similar optical density (OD_550_). Although the *P. phymatum* wild-type strain produced only a relatively low amount of biofilm, a statistically significant difference was observed with the *bceN* mutant that produced approximately two-fold less biofilm as compared to the wild-type (Figure 3), suggesting an important contribution of this BceN-dependent exopolysaccharide in biofilm formation. However, in the complemented mutant strain (*bceN*-COMP), which harbors an intact *bceN* gene in a plasmid construct, biofilm production was only partially restored, while the mutant strain containing the empty vector (*bceN*-pBBR) formed even less biofilm as the mutant strain (Figure 3).

### *P. phymatum* Produces the Polysaccharide Cepacian

**FIGURE 4:**
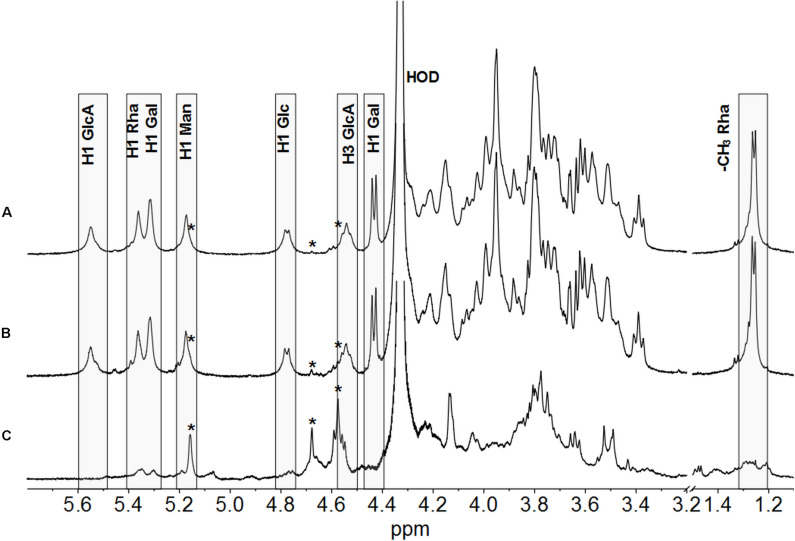
^1^H NMR spectra of the polysaccharides produced by *P. phymatum* wild type **(A)**, a *P. phymatum bceN* complemented mutant **(B)**, and a *P. phymatum bceN* mutant **(C)**. Spectra were recorded at 500 MHz and 70°C. Resonances attributed to the anomeric protons of CEP, residual water (HOD), H3 of glucuronic acid, and methyl group of rhamnose are reported. Boxes show diagnostic resonances of cepacian. H1, Anomeric proton; GlcA, Glucuronic acid; Rha, Rhamnose; Gal, Galactose; Man, Mannose; Glc, Glucose. Low intensity signals belonging to the polymer produced by the *bceN* mutant strain are indicated by stars.

A previous study from Ferreira et al. (2010) showed by Fourier transform infrared (FTIR) spectroscopy analysis that several *Burkholderia* strains, among these *P. phymatum*, produces a polysaccharide with structure similar to CEP’s produced by *Bcc* strains. In this study, we used NMR spectroscopy to analyze the polysaccharides synthetized by *P. phymatum* strain STM815 wild-type and mutant strains after 4 days of growth at 30°C on YE-mannitol plates (Cescutti et al., 2010). The polysaccharide was isolated and subjected to ^1^H NMR spectroscopy at 50°C. The ^1^H NMR spectrum of *P. phymatum* strain STM815 wild type (Supplementary Figure S2) showed very broad signals in the anomeric and ring regions of the spectrum, together with resonances attributable to methyl groups of acetyl substituents (at about 2.15 ppm) and 6-deoxy hexoses at 1.24 ppm. Integration of the areas under these peaks gave a ratio of 2.4 acetyl groups for each 6-deoxy hexose. The ^1^H NMR spectrum was highly similar to that of the polysaccharide CEP (Cescutti et al., 2000). With the aim of getting better resolved ^1^H NMR spectra, the polysaccharides produced by the three strains were sonicated, to decrease the viscosity of the solutions, de-acetylated and their spectra were recorded at 70°C (Figure 4). The ^1^H NMR spectrum of the polysaccharide produced by *P. phymatum* unambiguously identified it with CEP; assignments of resonances in the anomeric region and of the methyl group of rhamnose are reported in Figure 4 in agreement with published data (Cescutti et al., 2000). Comparison of the ^1^H NMR spectra of *P. phymatum* wild-type and *P. phymatum bceN*-COMP exopolysaccharide solutions (Figures 4A,B) showed their complete identity, thus demonstrating that also *P. phymatum bceN*-COMP strain produced CEP. Moreover, ^1^H NMR data of the *P. phymatum* wild type exopolysaccharide (Supplementary Figure S2) prior to deacetylation determined the presence of about 2.4 acetyl groups per repeating unit of CEP. The complemented strain also produced CEP with a comparable degree of acetylation (data not shown). On the contrary, although the ^1^H NMR spectrum of *P. phymatum bceN*-IM extract (Figure 4C) is compatible with that of a polysaccharide, it is completely different from the other two spectra, thus establishing that it does not produce CEP and nothing can be said about the structural identity of the polymer resorting only to this experimental data. Extremely low intensity signals belonging to the polymer produced by the *bceN*-IM strain are also present in the spectra of the other two samples, as indicated by stars in Figure 4. The data obtained suggested that CEP is the main polysaccharide produced by *P. phymatum* wild type in the experimental conditions used, while inactivation of the *bceN* gene led to lack of CEP biosynthesis, as expected.

### Cepacian Facilitates Plant Root Attachment

**FIGURE 5:**
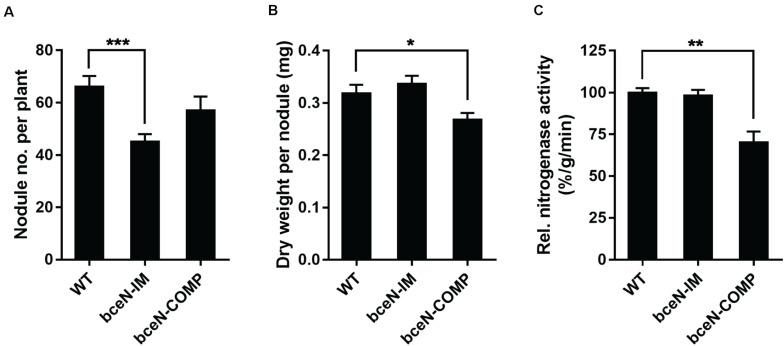
The symbiotic properties of *P. vulgaris* plants inoculated with *P. phymatum* wild-type (WT), *bceN* mutant (bceN-IM), and the complemented (bceN-COMP) strains. Number of nodules per plant **(A)**, dry weight per nodule **(B)**, and relative nitrogenase activity **(C)** were analyzed 21 dpi. The results are a combination of at least two independent experiments with two biological replicates of each strain, *n* = 16. Error bars indicate the standard error of the mean (SEM). ***p = 0.0007; *p = 0.0321; **p = 0.006.

**FIGURE 6:**
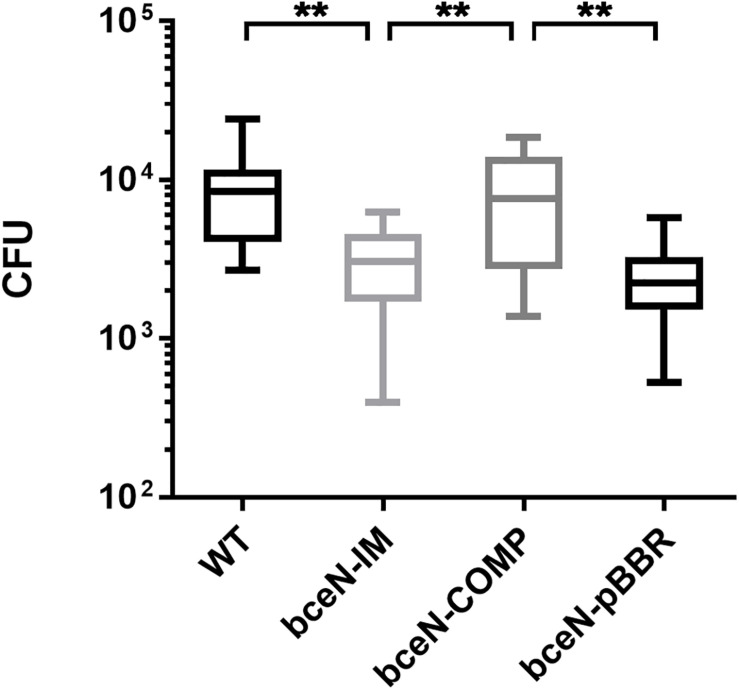
The *P. phymatum bceN* mutant is less efficient than the wild-type and the complemented strain in attaching to bean roots. Numbers of bacterial cells attached to bean roots after 4-h incubation followed by two stringent washing steps to remove loosely attached cells. Shown are five independent experiments with one biological replicate of each strain, *n* = 13. CFU, colony forming units. Error bars indicate the standard error of the mean (SEM). WT vs *bceN*-IM, **p = 0.0024; WT vs *bceN*-COMP, *p* = 0.8473; WT vs *bceN*-pBBR, **p = 0.0020; bceN-IM vs *bceN*-COMP, **p = 0.0040; *bceN*-COMP vs *bceN*-pBBR, **p = 0.0032.

To evaluate the potential role of CEP in the interaction of *P. phymatum* with plants, the symbiotic efficiency of the *bceN* mutant strain was tested using *Phaseolus vulgaris* var. Negro Jamapa as host plant. After 3 weeks of incubation in the green-house, several symbiosis-relevant parameters were assessed, i.e., nitrogenase activity, number and weight of nodules. While bean plants infected with the *bceN* mutant showed wild-type nitrogenase activity, the nodule number was reduced by around 30% (Figure 5). This observed reduction in nodule number could be partially complemented by introducing *bceN* on a plasmid. In contrast, the dry weight per nodule was similar in plants infected with mutant and wild type. However, nodules occupied with the complemented strain were lighter and displayed a 30% reduced nitrogenase activity as compared to wild-type nodules. This negative effect of the complemented strain in nitrogenase activity and nodules dry weight could be due to a possible overexpression of *bceN* from the plasmid. Additionally, a root attachment assay showed that after 4 h of incubation, the *bceN*-IM mutant as well as the empty vector control (*bceN*-pBBR) were impaired in attaching to germinated *P. vulgaris* roots relative to the wild type and the complemented strain (Figure 6). These results suggested a potential role of CEP in root attachment, the first step of root infection.

### Expression of the *bce* Cluster Is Induced Under Nitrogen Limitation and by Roots of Germinated Seeds

**FIGURE 7:**
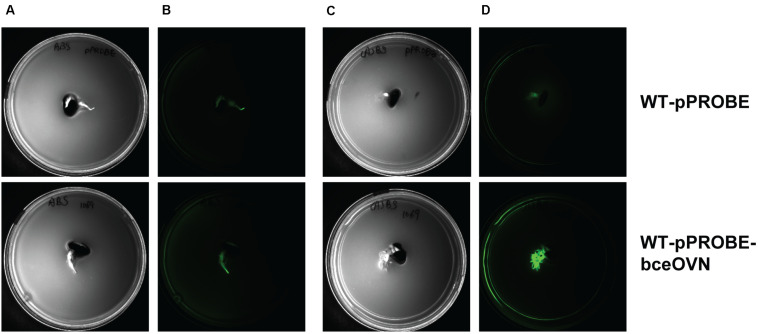
Expression of *bceN* is induced by the presence of a root of a germinated seed under nitrogen starvation. Images are bottom-up view of soft agar plates in the bright field **(A,C)** or under a GFP filter with 500 ms exposure **(B,D)**. Bacteria carrying either pPROBE vector without promoter (WT-pPROBE) or GFP driven by the promoter of *bceOVN* operon (WT-pPROBE-bceOVN) were grown in either ABS minimal medium **(A,B)** or (A)BS medium with limited nitrogen source **(C,D)** co-incubated with a root of a germinated seed for 48 h at 30°C. GFP was induced in WT-pPROBE-bceOVN cells located in the proximity of the bean root in nitrogen-limited medium.

Polysaccharide biosynthesis is a multi-step and complex process influenced by various environmental conditions such as the presence and amount of different nutrients (carbon, nitrogen, oxygen, and phosphate) (Serrato et al., 2006). Since there is a clear correlation between functional *bceN* and CEP production, we constructed a reporter strain where the promotor of the operon containing *bceN* was placed in front of the gene encoding the green fluorescent protein (*gfp*). GFP expression indicating *bceN* transcriptional activity was investigated under different conditions including nitrogen limitation, the presence of root exudates obtained from *P. vulgaris* and the presence of germinated seeds (Figure 7 and Supplementary Figure S3). Interestingly, both the presence of root exudates and nitrogen limiting conditions increased GFP expression by a factor of roughly two after 48 h incubation (Supplementary Figure S3). A maximal four-fold induction of expression was reached when the reporter construct was grown 48 h in nitrogen limiting conditions and in presence of root exudates (Supplementary Figure S3). When we incubated the *bce* reporter construct on a soft agar plate under nitrogen limiting conditions and in presence of a germinated bean seed, a clear induction of *bce* expression was observed (Figure 7). This result suggested that in addition to nitrogen limitation an inducing signal is present in the root exudates and/or on the root surface.

## Discussion

Rhizobial surface polysaccharides represent the interface between bacteria and roots in the soil and are therefore important traits for the establishment of a successful rhizobial symbiosis (Niehaus and Becker, 1998; Fraysse et al., 2003). Rhizobial polysaccharides are chemically diverse and species- and strain-specific. Several fast-growing rhizobia such as *S. meliloti* and *R. leguminosarum* synthetize exopolysaccharides containing octasaccharide repeating units of mainly glucose. The well-studied *S. meliloti* produces two types of exopolysaccharides, succinoglycan (*exo*/*exs* gene cluster), and galactoglucan (*wge*/*wga* gene cluster) (Gonzalez et al., 1996; Cheng and Walker, 1998; Pellock et al., 2000; Arnold et al., 2018).

In the recently discovered beta-rhizobia, nothing is known about the identity and the role of polysaccharides for persistence in soil and for symbiosis with legumes. Inspection of the complete genome of our model strain *P. phymatum* STM815 (Moulin et al., 2014) suggested the presence of nine potential polysaccharide biosynthetic gene clusters. However, none of these nine clusters displayed sequence similarity to the classical *exo*/*exs* and *wge*/*wga* clusters found in alpha-rhizobia, suggesting that beta-rhizobia use another exopolysaccharide to form biofilms on the plant root surface. *In silico* analysis of *P. phymatum* gene cluster (Bphy_1056–Bphy_1077) suggested that it is involved in the biosynthesis of CEP, a branched acetylated heptasaccharide also produced by many strains of the *Burkholderia sensu latu*, which are opportunistic pathogens (Conway et al., 2004; Cescutti et al., 2010; Ferreira et al., 2010; Hallack et al., 2010; Cuzzi et al., 2012). The role of CEP and particularly the associated switch to mucoid colonies in *Burkholderia* virulence is controversially discussed in the literature (Govan et al., 1993; Conway et al., 2004; Zlosnik et al., 2008, 2011; Silva et al., 2018). Recent data suggest that CEP is important for chronic infections and appears to be rather counter-productive for acute virulent infections (Silva et al., 2018). The fact that the *B. cenocepacia* ET12 epidemic CF isolates J2315, K56-2, and BC7 have lost their ability to produce CEP due to an 11 bp deletion in *bceB* (Moreira et al., 2003; Bartholdson et al., 2008) also suggests that CEP may not be required for acute infections caused by pathogenic *Burkholderia* strains.

In this study, we show for the first time a functional role of CEP for bacterial colonization of plants, which are the natural host not only for *Paraburkholderia* but also for several pathogenic *Burkholderia* strains (Coenye and Vandamme, 2003; Eberl and Vandamme, 2016). A *P. phymatum bceN* mutant did not produce CEP and was affected in biofilm formation. Moreover, when inoculated on common bean, the mutant led to the formation of a reduced number of root nodules (roughly 30% lower) and was affected in root attachment, suggesting that CEP increases the efficiency of *P. phymatum* to interact with its host plant. In contrast to the situation in *Bcc* strains and other *Burkholderia sensu stricto*, where the *bce*-I and *bce*-II clusters are separated by hundreds of kilobase pairs (Ferreira et al., 2010), the *bce* genes in *P. phymatum* STM815 are organized in one continuous gene cluster. Like in *P. phymatum*, other plant associated *Paraburkholderia* and *Caballeronia* strains previously belonging to the *Burkholderia* genus (*P. graminis* C4D1M, *P. xenovoran*s LB400, *P. phytofirmans* PsJN, *P. hospita* DSM 17164, *P. sprentiae* WSM5005, several *P. caribensis* strains, and *C. glathei* DSM 50014) also all harbor the *bce* genes in one single genomic locus (Ferreira et al., 2010) (Supplementary Figure S4).

A complex regulatory network controls CEP biosynthesis in response to changing levels of nutrients such as nitrogen, carbon and phosphate. We previously showed that in *P. phymatum* STM815 as well as in the opportunistic pathogen *B. cenocepacia* H111, the expression of the *bce* genes is significantly up-regulated in nitrogen limiting conditions (Lardi et al., 2015, 2017a). Moreover, in H111 *bce* expressions depends on the alternative sigma factor σ^54^ (RpoN) and on its activator NtrC (Lardi et al., 2015; Liu et al., 2017). In H111, a σ^54^ consensus sequence has been found in the *bce*-I and *bce*-II promoter regions suggesting that σ^54^ directly regulates *bce* expression. Cell-density dependent quorum sensing (QS) systems, which use LuxR-type transcriptional regulators and homoserine lactone signals (AHLs), were reported to regulate exopolysaccharide synthesis in different bacteria including *S. meliloti* (Gurich and Gonzalez, 2009). However, Coutinho et al. (2013) reported that in *P. phymatum* the BraRI AHL-dependent QS system represses expression of the *bce*-I cluster. Another important regulator of CEP biosynthesis is the response regulator OmpR. In the *Bcc* strain *B. multivorans*, OmpR was shown to be important for the mucoid to non-mucoid switch (Silva et al., 2018). Interestingly, several *Bcc* non-mucoid isolates produce large amounts of the polysaccharide CEP when tissue of the natural host plant onion was provided as nutrient source (Bartholdson et al., 2008). The plant compounds responsible for this induction of CEP biosynthesis resulted to be primarily sugar alcohols such as mannitol. Although we did not observe an activation of polysaccharide synthesis by mannitol in *P. phymatum* (Figure 2), the fact that plant metabolites induced CEP synthesis in *Bcc* strains, which also associate with plants, suggests that CEP may be primarily used for plant interaction rather than being a virulence factor in the pathogenic *Burkholderia* lineage.

In this study, we used a promoter fusion to elucidate activation of *bceN* expression under different growth conditions that mimic the natural soil environment of these bacteria (nitrogen limitation and presence of root exudates). We could observe a maximal activation of expression (four-fold after 48 h of incubation) when the cells were nitrogen-limited and incubated in presence of root exudates as well as on agar plates in presence of common bean roots suggesting that *P. phymatum* induces CEP synthesis in the often nitrogen limited rhizosphere. We speculate that the roots or its exudates present a signal, which induces *bce* expression. Interestingly, in the plant-growth promoting strain *Bacillus subtilis*, biofilm formation, an important feature for root colonization, was triggered by the plant polysaccharides arabinogalactan, pectin, and xylan. In addition, these plant polysaccharides were shown to be used as substrates for the synthesis of matrix polysaccharides of *B. subtilis* biofilms (Beauregard et al., 2013). Further studies are required to identify the signal(s) in the root exudates that induce CEP production. Whether CEP contributes to *P. phymatum*’s success in competitively colonizing different host plants and whether this represents a general feature for all plant-associated *Burkholderia* strains remain important open questions.

## Data Availability Statement

The raw data supporting the conclusions of this article will be made available by the authors, without undue reservation.

## Author Contributions

YL, PC, and GP conceived and designed the experiments. YL, SH, BB, and PC performed the experiments. YL, SH, LE, PC, and GP analyzed the data. YL, LE, PC, and GP wrote the manuscript. All authors contributed to the article and approved the submitted version.

## Conflict of Interest

The authors declare that the research was conducted in the absence of any commercial or financial relationships that could be construed as a potential conflict of interest.

## Supplementary Material

FIGURE S1 Aerobic growth of *P. phymatum* WT, *bceN*-IM, and *bceN*-COMP in liquid LB-NaCl **(A)** and ABS **(B)** media. Data of three biological replicates of each strain were combined.

FIGURE S2 ^1^H NMR spectrum of the polysaccharide produced by *P. phymatum* recorded at 500 MHz and 50°C. Main assignments are reported. The structure of CEP repeating unit is reported.

FIGURE S3 Expression of *bceN* is induced under nitrogen limiting growth conditions and in presence of root exudates after 24 and 48 h incubation. *P. phymatum* promoter reporter (WT-pPROBE-bceOVN) cells were incubated in four different minimal media: ABS, AB minimal medium with succinate as carbon source; (A)BS, ABS with limited nitrogen source; ABS + RE, ABS with root exudates; (A)BS + RE, (A)BS with root exudates and under nitrogen limitation. The GFP expression was normalized by cell density OD_600_. Statistical analysis was carried out on (A)BS + RE sample group in comparison with other conditions to show statistically significant induction of GFP expression in this group. Error bars indicate the standard error of the mean (SEM). ^****^*p* < 0.0001, ^∗∗∗^*p* < 0.001, and ^∗∗^*p* < 0.01.

FIGURE S4 Organization of *bce*-I and *bce*-II clusters in selected *Paraburkholderia* and *Burkholderia sensu stricto* strains (figure modified from Eberl and Vandamme, 2016, phylogenetic tree based on 16S rRNA).

TABLE S1 Bacterial strains, plasmids, and oligonucleotides used in this study.
