# Supplementary figures and images for: The Exopolysaccharide Cepacian Plays a Role in the Establishment of the Paraburkholderia phymatum – Phaseolus vulgaris Symbiosis
Source: Front Microbiol. 2020 Jul 16;11:1600. doi: 10.3389/fmicb.2020.01600 (PMC7378592; doi:10.3389/fmicb.2020.01600)

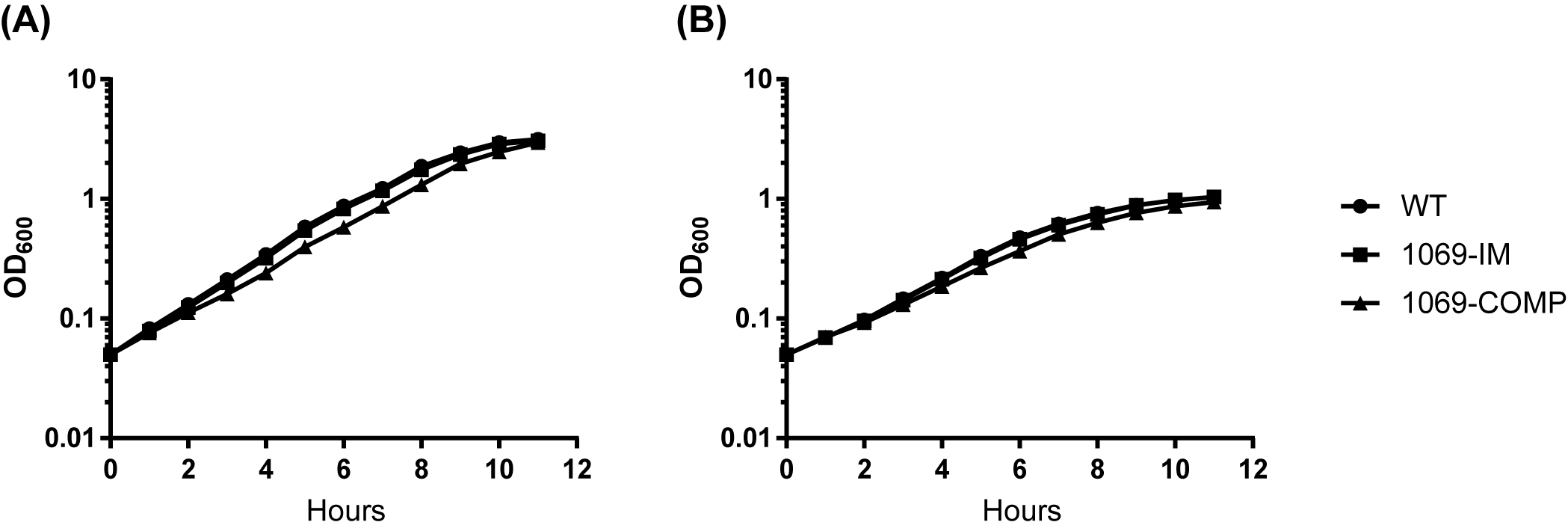

Supplement: FIGURE S1 — Aerobic growth of P. phymatum WT, bceN-IM, and bceN-COMP in liquid LB-NaCl (A) and ABS (B) media. Data of three biological replicates of each strain were combined. [file Image_1.TIF]

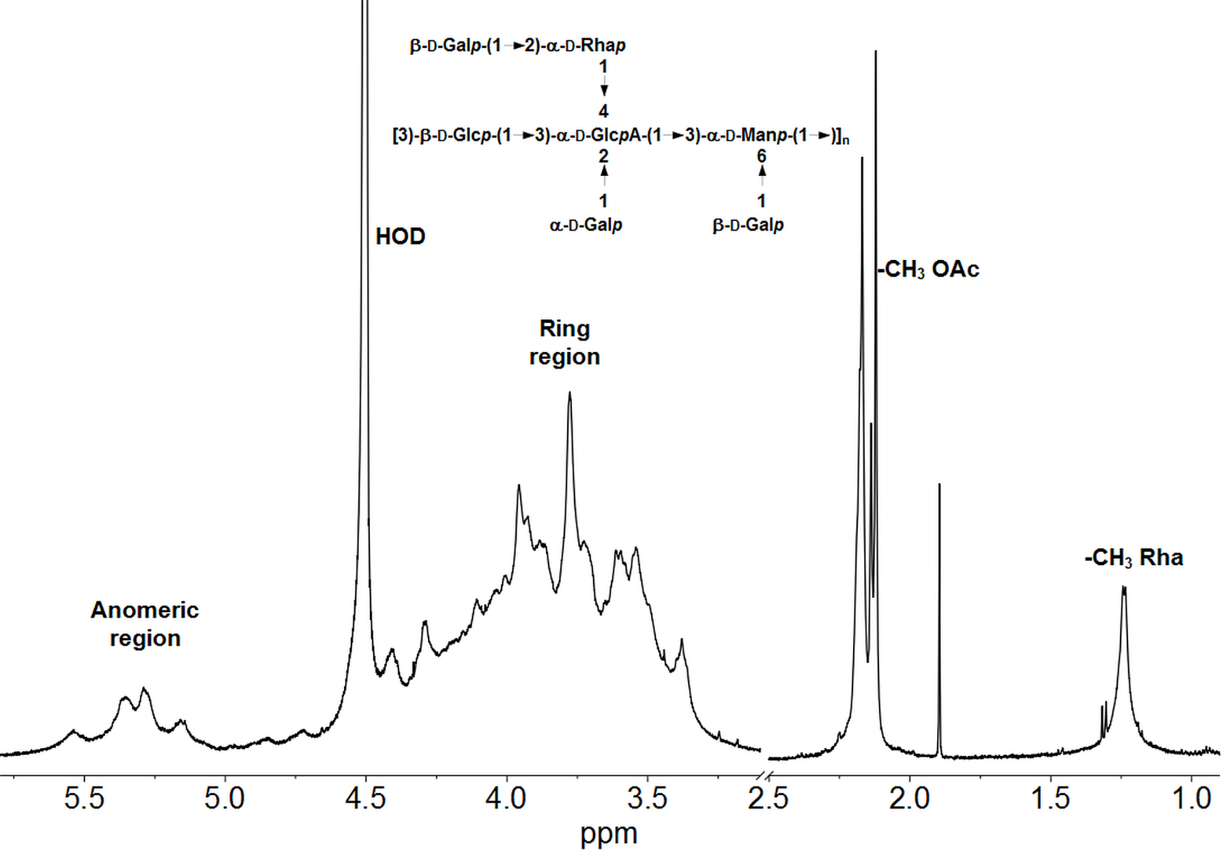

Supplement: FIGURE S2 — 1H NMR spectrum of the polysaccharide produced by P. phymatum recorded at 500 MHz and 50°C. Main assignments are reported. The structure of CEP repeating unit is reported. [file Image_2.TIF]

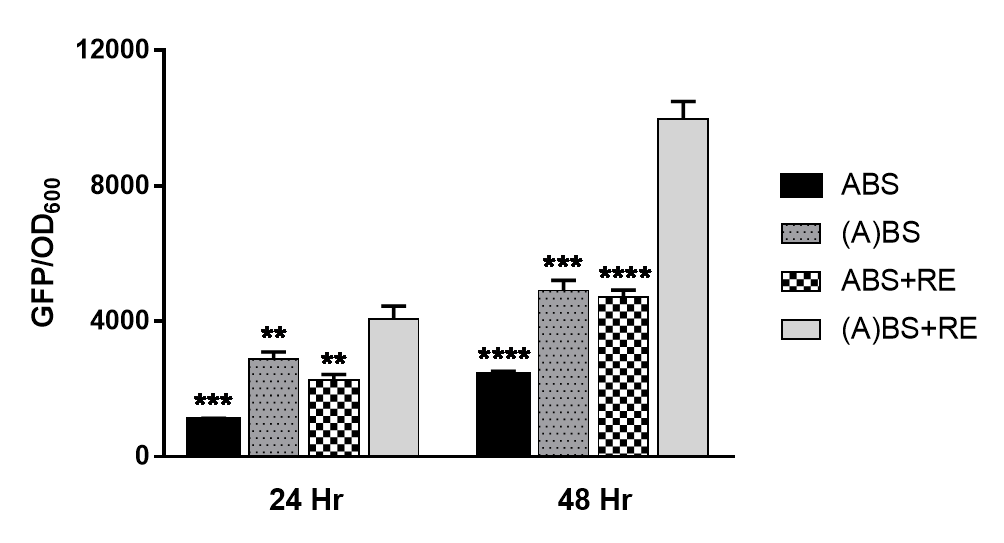

Supplement: FIGURE S3 — Expression of bceN is induced under nitrogen limiting growth conditions and in presence of root exudates after 24 and 48 h incubation. P. phymatum promoter reporter (WT-pPROBE-bceOVN) cells were incubated in four different minimal media: ABS, AB minimal medium with succinate as carbon source; (A)BS, ABS with limited nitrogen source; ABS + RE, ABS with root exudates; (A)BS + RE, (A)BS with root exudates and under nitrogen limitation. The GFP expression was normalized by cell density OD600. Statistical analysis was carried out on (A)BS + RE sample group in comparison with other conditions to show statistically significant induction of GFP expression in this group. Error bars indicate the standard error of the mean (SEM). ****p < 0.0001, ∗∗∗p < 0.001, and ∗∗p < 0.01. [file Image_3.TIF]

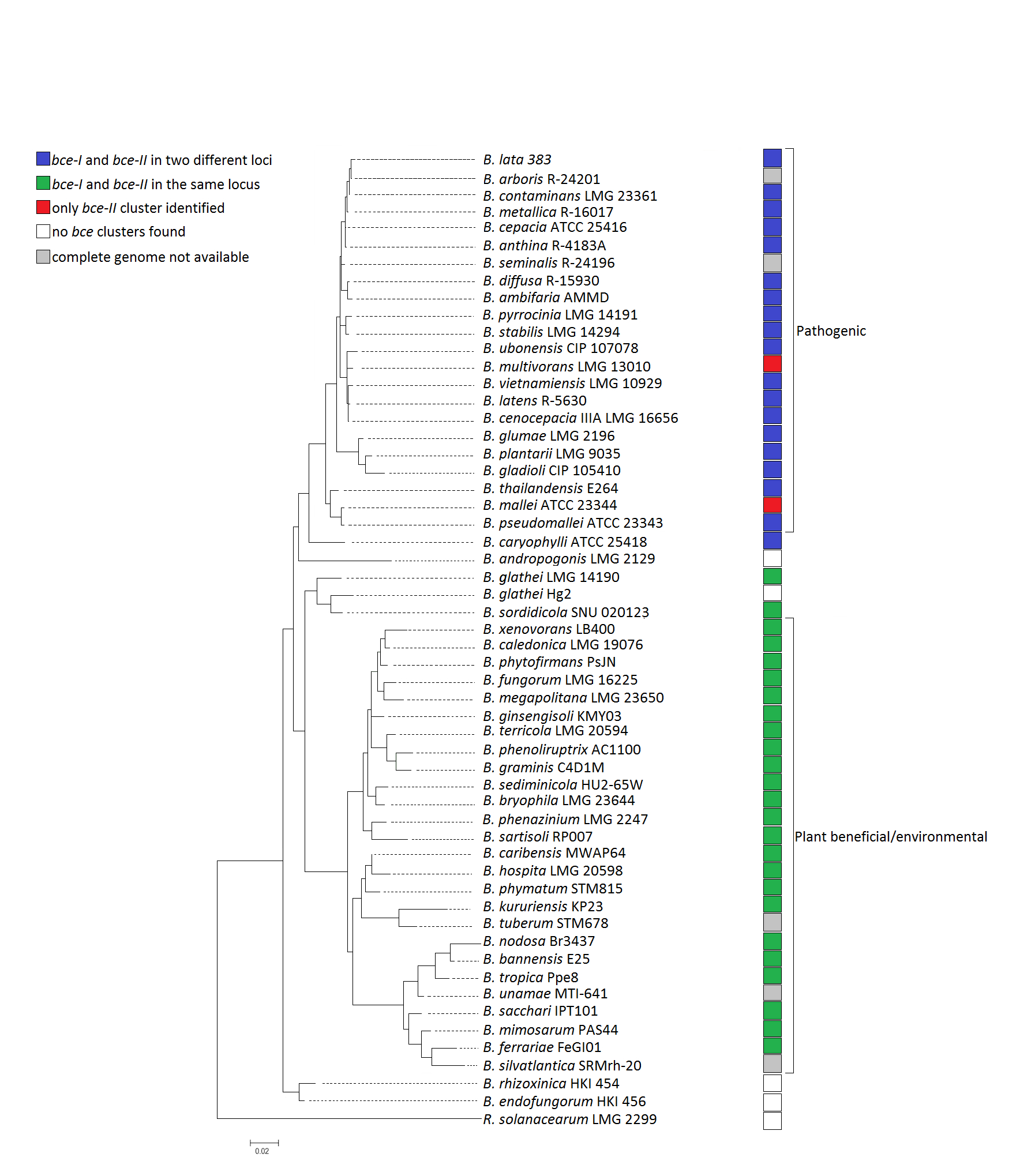

Supplement: FIGURE S4 — Organization of bce-I and bce-II clusters in selected Paraburkholderia and Burkholderia sensu stricto strains (figure modified from Eberl and Vandamme, 2016, phylogenetic tree based on 16S rRNA). [file Image_4.TIFF]
